# Validity of screening instruments for the detection of dementia and mild cognitive impairment in hospital inpatients: A systematic review of diagnostic accuracy studies

**DOI:** 10.1371/journal.pone.0219569

**Published:** 2019-07-25

**Authors:** Aljoscha Benjamin Hwang, Stefan Boes, Thomas Nyffeler, Guido Schuepfer

**Affiliations:** 1 Clinic for Neurology and Neurorehabilitation, Cantonal Hospital Lucerne, Lucerne, Switzerland; 2 Department of Health Sciences and Health Policy, University of Lucerne, Lucerne, Switzerland; 3 Staff Medicine, Cantonal Hospital Lucerne, Lucerne, Switzerland; University of Glasgow, UNITED KINGDOM

## Abstract

**Introduction:**

As the population ages, Alzheimer's disease and other subtypes of dementia are becoming increasingly prevalent. However, in recent years, diagnosis has often been delayed or not made at all. Thus, improving the rate of diagnosis has become an integral part of national dementia strategies. Although screening for dementia remains controversial, the case is strong for screening for dementia and other forms of cognitive impairment in hospital inpatients. For this reason, the objective of this systematic review was to provide clinicians, who wish to implement screening, an up-to-date choice of cognitive tests with the most extensive evidence base for the use in elective hospital inpatients.

**Methods:**

For this systematic review, PubMed, PsycINFO and Cochrane Library were searched by using a multi-concept search strategy. The databases were accessed on April 10, 2019. All cross-sectional studies that utilized brief, multi-domain cognitive tests as index test and a reference standard diagnosis of dementia or mild cognitive impairment as comparator were included. Only studies conducted in the hospital setting, sampling from unselected, elective inpatients older than 64 were considered.

**Results:**

Six studies met the inclusion criteria, with a total of 2112 participants. Diagnostic accuracy data for the Six-Item Cognitive Impairment Test, Cognitive Performance Scale, Clock-Drawing Test, Mini-Mental Status Examination, and Time & Change test were extracted and descriptively analyzed. Clinical and methodological heterogeneity between the studies precluded performing a meta-analysis.

**Discussion:**

This review found only a small number of instruments and was not able to recommend a single best instrument for use in a hospital setting. Although it was not possible to estimate the pooled operating characteristics, the included description of instrument characteristics, the descriptive analysis of performance measures, and the critical evaluation of the reporting studies may contribute to clinician's choice of the screening instrument that fits best their purpose.

## Introduction

### Background

Dementia is a progressive syndrome of global cognitive impairment. It encompasses a group of neurodegenerative disorders that are characterized by a progressive and irreversible decline of brain functions, with symptoms such as memory loss, disorientation, and the inability to perform daily activities of living independently [1]. Possible epiphenomena include neuropsychiatric symptoms and challenging behaviors of varying type and severity [2]. The most common dementia types include vascular dementia (VD), dementia with Lewy bodies (LBD), and Alzheimer's disease (AD), which is in around 40% the neuropathological diagnosis in patients with clinically diagnosed dementia disorder [3–6]. The process of AD pathology can be described as a continuum with a long asymptomatic preclinical stage; an early symptomatic clinical stage, which encompasses mild cognitive impairment (MCI), or prodromal AD; and a dementia stage, with dementia further divided into mild, moderate, and severe [7–9].

In 2015, more than 45 million people worldwide were estimated to be living with dementia. This number will almost double every 20 years, reaching 75 million in 2030 [10]. Of the Swiss population around 145 000 people are affected (year 2017), with a prevalence of 9% in individuals aged over 65 years, increasing to a prevalence of approximately 30% in adults aged 85 years and older [11]. Given that the prevalence of dementia rises steeply after the age of 65, the number of people living with dementia is expected to increase significantly due to the growing elderly population in Switzerland [12]. For the time being, Alzheimer's disease and related forms of dementia are incurable [13] and the burden on Swiss society is expected to grow substantially in the future. A national study, commissioned by the Swiss Alzheimer's Society, reported estimated annual costs of dementia at 6.3 billion CHF for 2007 and 6.96 billion CHF for 2009 [14, 15]. These findings are consistent with contemporary international studies, which predict rising global costs at similar rates until 2030 and beyond [10, 16, 17]. In response to this, Switzerland and many other countries have recognized dementia as a public health priority [18–20] and developed national dementia strategies [21].

Despite the absence of a cure for dementia, numerous strategies emphasize earlier diagnosis and intervention [22–25] in accordance with the Alzheimer Cooperative Valuation in Europe (ALCOVE), which recommends, that diagnosis should generally occur earlier than is currently common practice [26]. When speaking about earlier diagnosis, the conventional understanding usually distinguishes between early diagnosis and timely diagnosis. Whereas the term "early diagnosis" reflects the identification of people in the asymptomatic phase as a result of population or targeted screening, the term "timely diagnosis" is used to reflect diagnosis occurring at a time when patients and their family first notice changes in cognitive performance and seek medical examination [27]. In recent years, diagnosis has often been delayed or not made at all [22, 27, 28]. Multiyear delays from first symptom occurrence to presentation and inactivity by health professionals in offering help have been attributed mostly to patients or families not having the knowledge to realize the symptoms are part of a medical condition, the mutual false belief that nothing can be done, and the stigma of dementia preventing open discussions, respectively [27–30]. According to the World Alzheimer Report, only between a third and a half of people with dementia ever receive a formal diagnosis, that is usually necessary for insurers to pay for medical services [22, 27].

In view of this well-documented and widely recognized problem of inadequate recognition of dementia, national and international advisory- and policy-making groups have evaluated the possibility of earlier diagnosis facilitated by screening for dementia or mild cognitive impairment [31–37]. However, the U.S. Preventive Services Task Force concluded in its 2014 statement that evidence was insufficient to recommend routine screening for cognitive impairment in community-dwelling adults in the general primary care population who are older than 65 years [38]. Consistently, none of the remaining organizations recommended routine screening of patients in whom cognitive impairment was not symptomatic, but diagnostic workup when memory problems or dementia were suspected [36, 39].

This drive toward earlier diagnosis and intervention has been accompanied by a debate about the value of arriving at a diagnosis of dementia earlier in the disease process [27, 40–42]. Several studies reported evidence that supports a possible beneficial effect of early and accurate diagnosis [27, 28, 40]. Early diagnosis potentially offers the opportunity for early interventions that slow down or lessen the disease process [43–48], implementation of coordinated care plans while the patient is still competent to do so [49], better management of symptoms [50, 51], and postponement of institutionalization [47]. On the other hand, it should be acknowledged that diagnostic processes are costly and can come along with major psychological and psychosocial effects [27, 52–54]. Another concern is misdiagnosis, which can result in unnecessary or incorrect treatment [55].

Since then, new research findings regarding benefits and harms, the approval of new pharmaceutical agents for treatment, and growing media attention have converged to challenge this previous thinking about screening for cognitive impairment [56, 57]. As a result, changes in health care policies and priorities, such as the introduction of an opportunistic "dementia case-finding scheme" in the United Kingdom [58, 59], the Alzheimer's Foundation of America's National Memory Screening Program [60], and the implementation of cognitive assessments in the Medicare Annual Wellness Visit in the United States [61] have occurred.

### Rationale

Although most policy statements acknowledge that physicians should be sensitive to evidence of cognitive impairment and should act on their suspicion, recommendations for operationalizing the detection of possible dementia are scarce [35, 62]. Usually, frontline recognition and assessment of people with possible dementia, regardless of the setting, requires a test of cognitive function, third-party anamnesis, or both [38, 62]. At the moment, neuropsychological tests, usually developed and validated in primary care and memory clinics, are regarded as the most implementable instruments for screening [35, 36, 63, 64]. They are usually paper-and-pencil-based, easy to administer, and take between 10 and 45 minutes to complete. Country-specific guidelines and/or systematic literature reviews on which instruments to favor have already been published for the primary care setting [65–77]. With respect to the hospital setting however, where dementia and MCI are much more prevalent [78–80], comparable guidelines concentrate on minorities or selected patient groups, such as geriatric, stroke or emergency patients [81–84]. The variations in demographic features, health condition, disease prevalence, and severity but also, differences in test conditions (e.g. timing, interventions between index test and reference standard) entail separate external validation prior to general application. In response to this gap in information, two systematic reviews have recently been conducted to establish adequate tools for dementia screening, considering the particularities in secondary care. In 2010, Appels and colleagues [85] reported validation studies sampling from selected hospital outpatients with a focus on mild dementia and rather extensive screening instruments (10 to 45 min administration time). In comparison, in 2013, Jackson and colleagues [86] performed a review and meta-analysis of validated dementia screening instruments in unselected general hospital inpatients. Unselected, elective inpatients that account for some 40% of all hospitalizations have not been evaluated so far [87–89].

### Clinical role of index test

In the hospital setting, the knowledge that a patient has or might have dementia or MCI is essential because of the multiple immediate implications for care. Hospital medical staff may administer brief cognitive screening tests before or on the day of admission and, depending on the test results, cause additional investigations to be made to confirm whether a diagnosis is present or not; provide appropriate care during the hospital stay (e.g., choice of anesthesia, involvement of primary caregiver, medication management, etc.), and realize adequate discharge management [90–93], which may then lead to avoiding new medical events known to be more likely among patients with cognitive impairment and promoting earlier diagnosis [94, 95].

### Objective

Many screening instruments are recommended for the application in primary care setting but not so many, for screening in older hospital inpatients. The aim of this review is to provide clinicians, who wish to implement screening for dementia or MCI, an up-to-date choice of practical and accurate instruments that have been validated well for the use in unselected, elective hospital inpatients.

## Methods

### Eligibility criteria

Articles were limited to the English and German languages. Abstracts fulfilling the following criteria were included:

Clinical setting: Only studies conducted in a hospital setting (general or university hospital) involving elective inpatients over 64 years of age as the main study group, or as a clearly defined subgroup, were included. The aim of the review was to identify screening instruments and to establish their diagnostic accuracy in unselected samples within the hospital setting. For this reason, studies including participants that were selected on the basis of a specific disease or medical field (e.g., Parkinson's disease or orthopedic patients) were excluded. In addition, wards providing services exclusively for patients with diseases related to dementia (psychiatric and neurology) were excluded. In case of mixed settings, studies were excluded if no separate data was presented for outnumbered elective inpatients.

Target condition: Mild cognitive impairment (MCI), dementia, and any common dementia subtype, including Alzheimer's disease (AD), vascular dementia (VD), Lewy body dementia (LBD), and frontotemporal dementia (FTD).

Index tests: Screening during pre-operative examination or hospitalization in the more stable, elective inpatients might be less affected by time as a limiting factor. In comparison, especially in emergency departments or primary care setting, where time is scarce, administration time is key determinant of whether screening instruments are used in clinical practice or not [96, 97]. For this reason, screening instruments with a short, but also medium administration time (up to 15 minutes in non-impaired patients) were considered. Furthermore, instruments had to cover more than one cognitive domain to be eligible for inclusion because the coverage of multiple cognitive domains undoubtedly increases the instrument's sensitivity to different types of dementia [96, 98]. Optimally, the instrument had to cover at least the domains of "learning and memory" and "executive function", which are considered central to a diagnosis of dementia -most particularly to its most prevalent forms, Alzheimer's disease (AD) and vascular dementia (VD) [3, 99, 100].

Although the incorporation of informant reports into assessments for dementia is known to increase the overall accuracy of detection of cases and non-cases, tests that are wholly informant rated were not considered [101–103], solely because, in the clinical setting, the presence of an informant is not the norm and proxy rating comes with confidentiality concerns. Self-administered tests, measures that assessed daily living activities and functional status, and telephonic or computerized self-tests were also excluded.

The full-texts were reviewed against the following additional inclusion criteria:

Types of studies: Cross-sectional studies, in which inpatients received the index test and reference standard diagnostic assessment during a hospital stay, preferably on the day of admission and before the commencement of treatment, were included. Studies were excluded for inadequate reporting (e.g., studies that did not report sensitivity or specificity), non-availability of the full-text article, or if subjects with prevalent target disease at baseline were included. Case-control studies and longitudinal studies (or related, nested case-control studies) were excluded due to the high risk of spectrum bias [96]. Also, studies sampling fewer than 100 participants were excluded due to the potential for bias in selection and lack of representativeness.

Reference standards: Studies were included that used a reference standard for MCI, all-cause dementia or any standardized definition of subtypes. For MCI, the reference standard diagnosis had to be made according to published criteria, that is, Diagnostic and Statistical Manual of Mental Disorders, Fifth Edition (DSM-V) [104], National Institute of Ageing-Alzheimer's Association criteria [105], Petersen [106], Gauthier [107], or Winblad [108] criteria. For all-cause dementia, any version of the DSM [104, 109], and the International Classification of Diseases (ICD) [110] criteria were included. For dementia subtypes (e.g. AD or probable AD, vascular dementia, or Lewy body dementia) common diagnostic criteria were included [111–114]. In order not to further restrict the number of eligible studies, diagnostic accuracy studies that compared the index test with a diagnosis based on an expert consensus, or results of the Mini-Mental State Examination (MMSE) test were also included. Studies that applied a neuropathological diagnosis which needs to be verified post-mortem were excluded [115].

### Information sources

An electronic literature search was conducted in the following databases: PubMed from 1972, Cochrane Library from 1992, and PsycINFO from 1967. All databases were accessed on the September 6, 2018. To ensure published literature saturation relevant systematic literature reviews, the reference sections of selected articles and the 'similar articles' feature in PubMed were assessed for further relevant studies. An update search was performed on April 10, 2019.

### Search

To search the databases, a multi-concept search strategy was applied. The primary strategy used the following concepts: (a) Disease: Dementia and cognition disorders (general terms, both free text and MeSH, exploded); (b) Outcome: Validation and sensitivity and specificity values (both free text and MeSH, exploded); (c) Intervention: Diagnostic tests, mass screening, etc. (both free text and MeSH, exploded), and (d) Setting: Aged in-patients (both free text and MeSH, exploded).

The secondary strategy involved a review of dementia practice guidelines [35] to identify recommended screening instruments. Irrespective of the setting targeted by those practice guidelines, recommended instruments were used as key search terms to run an additional electronic search if they met the fore mentioned criteria of a screening instrument. The original search strategies were developed for the PubMed database and slightly adapted to run on Cochrane Library and PsychINFO. The search strategy was peer-reviewed (by MA), using the PRESS 2015 Guideline Evidence-Based Checklist [116]. Disagreements were discussed and decided by consensus. The search strategy for PubMed is documented in the Supporting Information (S1 Appendix). Search strategies for PsychINFO and Cochrane are available from the corresponding author upon request.

### Study selection

The titles and abstracts (where needed) were independently screened by the author (ABH) and one trained assessor (MG). Full-texts were independently reviewed by two assessors (ABH and MG). Any disagreements were discussed and decided by consensus. For all articles whose full-text was screened, additional information from authors was sought to resolve questions about eligibility, and reasons for exclusion were recorded (maximum three email contact attempts; if data was not available, the article was excluded). All articles selected were included only after reaching a consensus among all the authors. The study selection process was detailed in a Preferred Reporting Items for Systematic Reviews and Meta-Analyses (PRISMA) flow diagram.

### Data extraction and management

From the selected articles, the following data was extracted using an extraction sheet, which was pilot-tested on three randomly selected articles: Country; type of hospital; patient group; target condition; sample size; age, and mean age; gender ratio; index test and applied cut-off; reference standard; point in time of screening; other assessments; assessment for delirium; prevalence, and sensitivity and specificity. The complete data extraction form is available on reasonable request from the corresponding author. Data extraction was done by one author (ABH) and one reviewer (MG). Any disagreements were decided by consensus. In case of uncertainties, study authors were contacted by e-mail (maximum of three email attempts; if data was not available, the article was tagged with a no-data-badge).

### Risk of bias and applicability

One author (ABH) and one reviewer (MG) independently assessed, discussed, and reached a consensus on the methodological quality of each included study, using the recommended quality assessment tool for Diagnostic Accuracy Studies (QUADAS-2) [117, 118] and the Standards for the Reporting of Diagnostic Accuracy studies checklist (STARD 2015) [119]. Brief definitions describing the operational application of both instruments are detailed in the Supporting Information (S2 and S3 Appendices).

### Statistical analysis and synthesis of results

Statistical analysis was performed according to the Cochrane guidelines for diagnostic test accuracy reviews [118]. For all included studies, diagnostic accuracy data was presented in two-by-two tables and used to calculate sensitivity and specificity values as well as measures of statistical uncertainty (95% confidence intervals). Data from each study was presented graphically by plotting estimates of sensitivities and specificities on a coupled forest plot. For studies that reported more than one threshold, only sensitivity and specificity data at the most common threshold were included in the two-by-two table. Investigation of heterogeneity was done through visual examination of the forest plot.

### Registration and protocol

The pre-defined review protocol was registered at the PROSPERO international prospective register of systematic reviews (https://www.crd.york.ac.uk/prospero/, registration number CRD42019133093). The protocol for this review and its primary search strategy are accessible on https://www.protocols.io/ and as supporting information; see S1 and S4 Appendices.

## Results

### Study selection and characteristics

**Fig 1:**
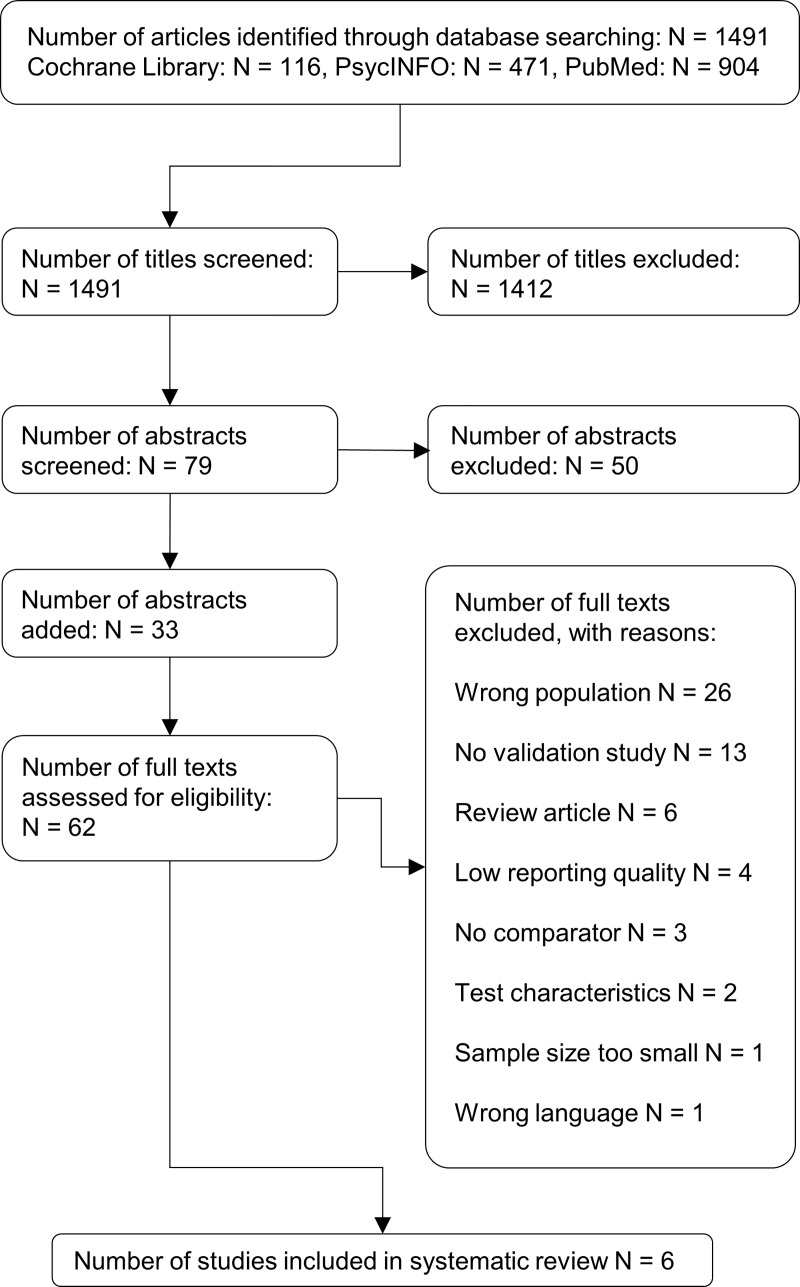
Study flow diagram.

The initial literature search revealed 1524 citations. Thirty-three of them originated from the reference sections of selected systematic reviews. In the end, 1518 articles were excluded and six studies investigating the validity of five different instruments were included in the reviewing process. The results of the search are summarized in the flow diagram (Fig 1. Study flow diagram). The inter-rater agreement between the author (ABH) and the peer-reviewer (MG) was moderate with a Cohan's kappa of 0.48

Of the six studies, two studies were conducted in Australia and one study each took place in the Netherlands, Switzerland, the UK, and the US. All studies sampled from consecutive inpatients being admitted to university hospitals [120–122], general hospitals [123], or to a mix of both [124, 125]. While five studies included inpatients from medical and surgical wards, one study included inpatients from the general internal medicine ward only [122]. Merely two studies reported admission or discharge diagnoses [120, 122]. Also, reporting of patient characteristics (ethnicity, marital status, living situation, and educational attainment) was inconsistent. Diagnosis included all-cause dementia (4) and cognitive impairment (2), as defined by the third and fourth DSM version, [121, 123, 125] an expert diagnosis following interview and cognitive assessment [126]; and MMSE test results as criterion standard, respectively [122, 124]. Delirium as a cause of cognitive impairment was ruled out in four studies [121, 123, 125, 126]. Studies ranged in size from 103 to 776 participants (total n = 2112), including patients older than 69 years, with a mean value between 78 and 80 years. On average, studies included slightly more women (58%) than men (42%). The mean point prevalence of the target condition ranged between 14% and 33%. Further study details, that is, index test, cut-off value, point in time of screening, or other performed assessments can be found in the Characteristics of Included Studies (Table 1).

**Table 1:** Characteristics of included studies.

Study	Setting	Target condition	Sample size	Age, mean age (SD)	Sex, female %	Education	Index test	Cut-off	Reference standard	Moment of screening	Other assessments	Delirium assessment	Prevalence % ***
Death et al. (1993)	UK, 2 sites General Hospitals, medical & surgical wards	Dementia	117	>70, 79 (SD = 6*)	59	No data	CDT	Clock class 1 & 2	DSM-III	Within 48 hrs. of admission	MMSE, psychological and physical examination	Yes	27
Inouye et al. (1998)	US, 1 site University Hospital, medical & surgical wards	Dementia	776	>69, 78 (SD = 6.1)	55	mean 11.3 years	T&C	≥1 error	Panel decision based on interview, assessment & medical record data	During hospitalization	Story Recall Test, Visual Analog Scale for Confusion, self-reported ADLs, Standard Near-Vision Test, Modified Blessed Dementia Rating Scale and MMSE	Yes	14
Nair et al. (2007)	Australia, 1 site University Hospital medical & surgical wards	Dementia	103	>69, 80 (SD = 6.9)	64	20% no education; 34% primary education; 38% incomplete high school education; 9% completed high school; 2% tertiary education	T&C	≥1 error	DSM-IV	72 hrs. after admission	Telling Time Task, Making Change Task and MMSE	Yes	33**
							MMSE	23/24					
Travers et al. (2013)	Australia, 4 sites University & General Hospitals, medical & surgical wards	Dementia	462	>69, 80 (SD = 6.5)	57	82% had at least achieved secondary level education	CPS	≥2	DSM-IV	Within 48 hrs. of admission (or 72 hours after surgery)	ADL, IADL, interRAI Acute Care, MMSE, CAM, 16-item IQCODE	Yes	18
							MMSE	23/24					
Büla et al. (2009)	Switzerland, 1 site University Hospital medical wards only	Cognitive Impairment	401	>74, 82 (SD = 5)	61	46% had less than high school	CPS	≥2	MMSE	Within 48 hrs. of admission	ADL, Geriatric Depression Scale, Charlson Comorbidity Index	No	32
Tuijl et al. (2012)	Netherlands, 2 sites University & General Hospital, medical & surgical wards	Cognitive Impairment	253	>69, 80 (SD = 6.7)	56	41% had less than 11years of education; 34% had more than 12 years	6CIT	≥11	MMSE	Within the first 4 days of the stay/during preoperative screening	-	No	28

(A) Abbreviations: Prev.: Prevalence; SD: Standard Deviation; CDT: Clock Drawing Test; DSM-II/IV: Diagnostic and Statistical Manual of Mental Disorders II/IV; MMSE: Mini Mental Status Examination; T&C: Time & Change Test; ADLs: Activities of Daily Living; CPS: Cognitive Performance Scale; 6CIT: 6 Item Cognitive Impairment test; IADL: Instrumental Activities of Daily Living; CAM: Confusion Assessment Method; IQCODE: Informant Questionnaire on Cognitive Decline in the Elderly

(B) *SD has been approximated

(C) ** Includes DSM IV diagnosis of dementia or delirium

(D) *** according to reference standard

(E) Funding sources: Death et al.–No data; Inouye et al.: This work was supported in part by grants from the National Institute on Aging, from the Commonwealth Fund and from the Retirement Research Foundation; Nair et al.: No data; Büla et al.: This work was supported by a grant from the Public Health Service, Canton de Vaud, Switzerland; Tuijl et al.: No outside sources of funds; Travers et al.: This research was funded by a National Health and Medical Research Council (NHMRC) Project Grant (ID: 511125).

### Risk of bias and applicability

**Fig 2:**
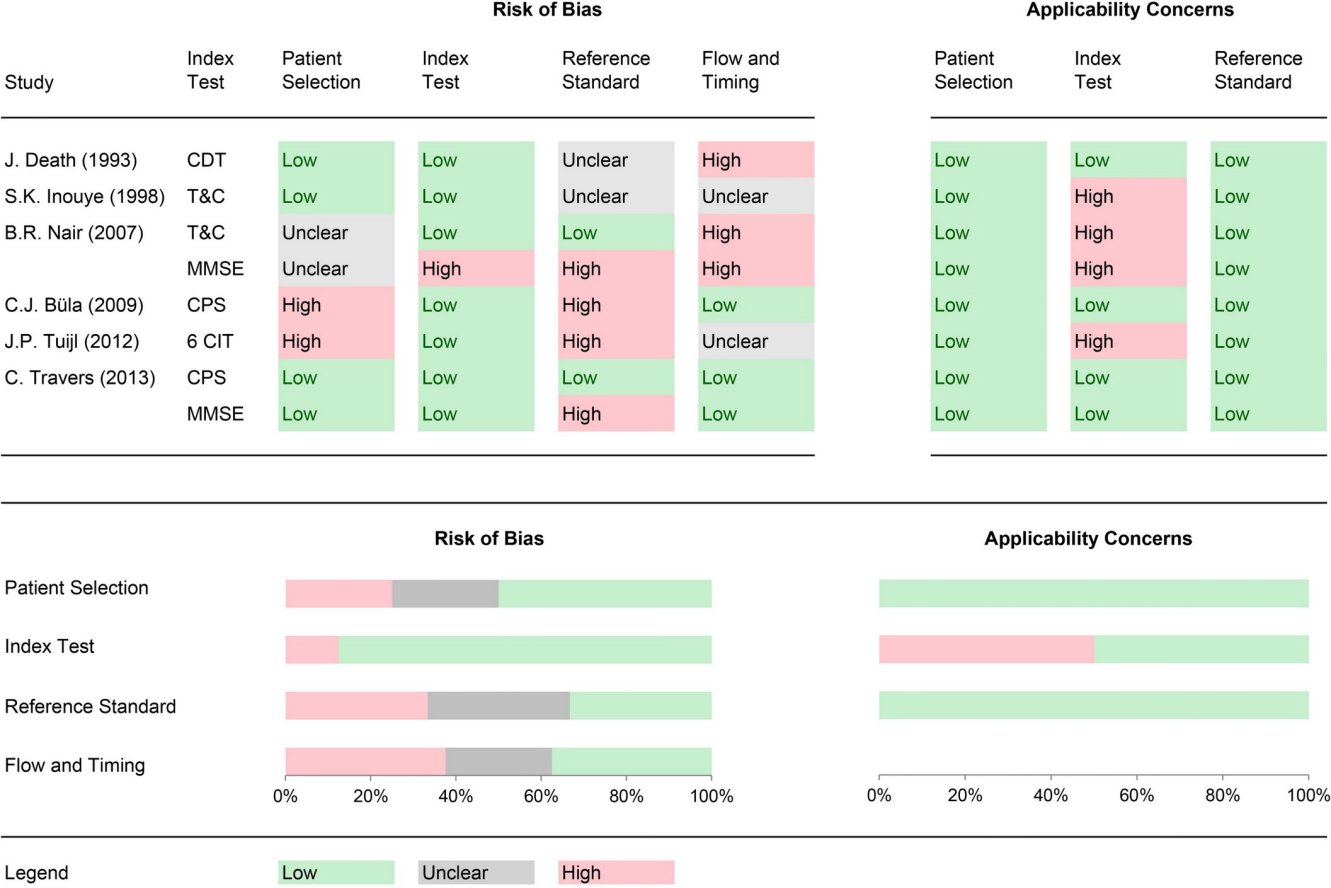
Risk of bias and applicability concerns graph. (A) Abbreviations: CDT: Clock Drawing Test; T&C: Time & Change Test; MMSE: Mini-Mental Status Examination; CPS: Cognitive Performance Scale; 6CIT: Six-Item Cognitive Impairment test.

To assess the study quality and risk of bias, all included studies were reviewed using the QUADAS-2 methodology (Fig 2. Risk of bias and applicability concerns graph). Of all included studies, only Travers and colleagues [125] Cognitive Performance Scale (CPS) was rated at low risk in all the categories. In the patient selection domain, two studies were rated high risk of bias due to inappropriate exclusion of privately insured and comatose patients [122] and patients who were not able to sustain their attention sufficiently [124]. Two studies were considered as having an unclear risk because it was not stated whether a consecutive or random sample was enrolled [121]. In the index test domain, one study was rated at high risk of bias because the index test results were interpreted with the knowledge of the results of the reference standard [121]. In the reference standard domain, four studies were rated high risk, and two studies were considered to be at unclear risk of bias. The high risk-rated studies interpreted the reference standard results with the knowledge of the results of the index test [121, 125] or used a reference standard that is not likely to correctly classify the target condition [122, 124]. The studies considered as unclear only mentioned vague information about whether the reference standard rater was blinded to the results of the index test results or not [123, 126]. In the flow and timing domain, three studies were rated at high risk of bias because not all patients were included in the analysis [121] or not all patients received the same reference standard [123]. Two studies were rated with unclear risk of bias. They did not provide enough information about whether any interventions were done between the administration of the index test and the reference standard [124, 126].

Finally, regarding the assessment of applicability concerns, for the majority of studies there was no concern that the included patients, the conduct and interpretation of the index test, or the reference standard did not match the review question. However, for four studies there were high applicability concerns, because the index tests took place more than 48 hours after hospital admission and there were no sufficient information whether prior index testing any measures with potentially negative effects on patient's cognitive performance were performed or not [121, 124, 126].

Reporting quality was assessed using the STARD guideline. In all included articles limitations in reporting were found. Reporting items of particular concern were: Description of sample size calculation (Item 18: No paper did report on its pre-specified sample size), flow of participants (Item 19: No paper did visualize the patient flow using a flow diagram), distribution of alternative diagnoses in those without the target condition (Item 21b: No paper did establish and document diagnoses of subjects without the target condition) and reporting of registration number and name of registry (Item 28: Only one study reported on this item). Affected by irregular reporting were also items 15 and 16, describing how indeterminate index test or reference standard results and missing data were handled (only two studies reported on these items). Further details are illustrated in Table 2. (STARD 2015 Checklist).

**Table 2:** STARD 2015 checklist.

			Study					
Item	Section	STARD 2015 Checklist Criteria	J. Death (1993)	S.K. Inouye (1998)	B.R. Nair (2007)	C.J. Büla (2009)	J.P. Tuijl (2012)	C. Travers (2013)
1	Title or Abstract	Identification as a study of diagnostic accuracy using at least one measure of accuracy (such as sensitivity, specificity, predictive values or AUC)	2	2	2	2	2	2
2	Abstract	Structured summary of study design, methods, results and conclusions (for specific guidance, see STARD for Abstracts)	-1	2	2	1	2	2
3	Introduction	Scientific and clinical background, including the intended use and clinical role of the index test	2	2	2	2	2	2
4	Introduction	Study objectives and hypotheses	2	2	2	2	2	2
5	Methods	Whether data collection was planned before the index test and reference standard were performed (prospective study) or after (retrospective study)	2	2	2	2	2	2
6	Methods	Eligibility criteria	2	2	2	2	2	2
7	Methods	On what basis potentially eligible participants were identified (such as symptoms, results from previous tests, inclusion in registry)	2	2	2	2	2	2
8	Methods	Where and when potentially eligible participants were identified (setting, location and dates)	1	2	1	1	2	2
9	Methods	Whether participants formed a consecutive, random or convenience series	2	2	0	2	2	2
10a	Methods	Index test, in sufficient detail to allow replication	2	2	2	2	2	2
10b	Methods	Reference standard, in sufficient detail to allow replication	2	2	2	2	2	2
11	Methods	Rationale for choosing the reference standard (if alternatives exist)	2	1	2	1	2	2
12a	Methods	Definition of and rationale for test positivity cut-offs or result categories of the index test, distinguishing pre-specified from exploratory	2	2	2	2	2	2
12b	Methods	Definition of and rationale for test positivity cut-offs or result categories of the reference standard, distinguishing pre-specified from exploratory	2	2	2	2	2	2
13a	Methods	Whether clinical information and reference standard results were available to the performers or readers of the index test	2	2	2	2	2	2
13b	Methods	Whether clinical information and index test results were available to the assessors of the reference standard	0	0	2	2	2	2
14	Methods	Methods for estimating or comparing measures of diagnostic accuracy	2	2	2	2	2	2
15	Methods	How indeterminate index test or reference standard results were handled	2	-1	-1	2	-1	2
16	Methods	How missing data on the index test and reference standard were handled	0	2	-1	-1	-1	2
17	Methods	Any analyses of variability in diagnostic accuracy, distinguishing prespecified from exploratory	-1	2	-1	2	2	1
18	Methods	Intended sample size and how it was determined	-1	-1	-1	-1	-1	-1
19	Results	Flow of participants, using a diagram	-1	-1	-1	-1	-1	-1
20	Results	Baseline demographic and clinical characteristics of participants	1	2	1	2	2	2
21a	Results	Distribution of severity of disease in those with the target condition	2	2	1	2	-1	2
21b	Results	Distribution of alternative diagnoses in those without the target condition	-1	-1	-1	-1	-1	-1
22	Results	Time interval and any clinical interventions between index test and reference standard	2	0	0	0	1	2
23	Results	Cross tabulation of the index test results (or their distribution) by the results of the reference standard	2	2	2	-1	2	2
24	Results	Estimates of diagnostic accuracy and their precision (such as 95% CIs)	1	2	2	2	2	2
25	Results	Any adverse events from performing the index test or the reference standard	-1	2	2	2	2	-1
26	Discussion	Study limitations, including sources of potential bias, statistical uncertainty and generalizability	1	2	2	2	2	2
27	Discussion	Implications for practice, including the intended use and clinical role of the index test	1	2	2	2	2	2
28	Other Information	Registration number and name of registry	-1	-1	-1	-1	-1	2
29	Other Information	Where the full study protocol can be accessed	2	2	2	2	2	2
30	Other Information	Sources of funding and other support; role of funders	-1	2	-1	2	2	2
Sum of all reporting items			35	48	37	45	46	55

(A) Legend: 2 = fully reported, 1 = partially reported, 0 = unclear, -1 = not reported/missing

### Findings

For the final review, six studies on five unique screening instruments were selected [121–126]. The instruments studied were the Clock- Drawing Test (CDT), the Cognitive Performance Scale (CPS), the Mini-Mental Status Examination (MMSE), the Time & Change (T&C) test, and the Six-Item Cognitive Impairment Test (6-CIT). The MMSE and T&C were administered in two studies [121, 122, 125]. Hereinafter, all five instruments are briefly described, following a portrayal of the diagnostic accuracy data in the setting to be evaluated.

#### Instruments

Clock Drawing Test (CDT). The CDT is a commonly used, brief neuropsychological test, sensitive to cognitive changes and functional skills [127]. Originally, the CDT was developed as an instrument for attentional and visual disorders [128, 129]. Due to its valuable characteristics (i.e., free of charge, quick, easy to administer, relatively high robustness), the CDT has gained in popularity among practitioners and researchers as a screening instrument for Alzheimer's dementia either by itself or as part of a test battery [130–137]. Because of its simplicity and brevity, the CDT is well accepted by older and very old adults [138]. Although the CDT covers several cognitive domains and can thus provide some more information on the actual nature of cognitive impairment, it does not differentiate among Alzheimer's disease (AD), dementia with Lewy bodies (DLB), and cognitively impaired Parkinson's disease [139, 140]. In clinical practice, there are basically three approaches on how to administer the CDT. The most common administration instructions ask the patient to draw a clock face with all its numbers and set the time to 10 past 11 [137]. Variations can include a pre-drawn clock face, a different time setting, or a toy clock from which the patient needs to read the time [132, 141, 142]. In addition to the differences in how to administer the test, there are also various scoring methods [137, 143]. Commonly put into practice is the classification of drawn clocks into distinct classes. Death and colleagues distinguishes four classes, normal clocks (4), clocks with minor spacing abnormalities (3), clocks with major spacing abnormalities (2) and bizarre clocks (1). Clocks class 1 and 2 indicate cognitive impairment, and class 3 and 4 no cognitive impairment [123]. In the literature, there is no consensus about which scoring method is the most adequate, mainly because comparative studies have been questioned with respect to a methodologically diverse set of included studies [144].

Cognitive Performance Scale (CPS). The Cognitive Performance Scale was developed in 1994 as a standardized, comprehensive assessment instrument for cognitive function in nursing-home residents [145]. It is based on a subset of five items of the Minimum Data Set (MDS), which were combined to create a single, functionally meaningful, seven-category ranked scale [145]. The CPS is free of charge and takes less than 3 minutes to administer and covers several cognitive subdomains (i.e. short- and long-term memory, orientation, and executive function) [145]. For scoring, all items are combined by a branching logic with five decision nodes, daily decision making ability, short-term memory, procedural memory, ability to make self-understood and ability to feed oneself. Using this branching logic, patients can be classified in seven ranked categories, ranging from Intact (0) to Very Severe Impairment (6) [145]. According to Morris and colleagues, a rank of 2 or higher indicates the presence of cognitive impairment.

Due to limitations, that is, low sensitivity to early impairment, overestimation in dependent patients with comorbidities and depressive symptoms and underestimation in older patients, Morris and colleagues revised the CPS in 2016 [146]. The revised Cognitive Performance Scale 2 includes a new, most-independent category and a series of dichotomous severity options, providing a stepped hierarchical report of cognitive performance decline. The levels of cognitive impairment expanded from seven to nine and, thus, enabled repeated assessments to detect changes, in particular in early levels of cognitive decline.

At present, both CPS versions can only be scored following the administration of the complete interRAI AC, an instrument to obtain detailed information about patient's physical and cognitive status and psychosocial functioning (including the Minimum Data Set).

Mini-Mental Status Examination (MMSE).The Mini-Mental Status Examination is a brief measure of cognitive functioning and its change and was developed more than 30 years ago [136]. Although originally distributed free of charge, the MMSE has recently been subject to copyright restrictions [147]. The MMSE takes around five to 10 minutes to administer and is available in multiple languages. Its use as a cognitive test is widespread among researchers and specialists [148–150], though it is not very popular in primary care, because its administration time is considered too long [151]. The MMSE was developed from items selected from different neuropsychological batteries. Although it covers five cognitive subdomains, Orientation, Registration, Attention and Calculation, Recall, and Language [136], the MMSE is not an adequate instrument to identify early stages of dementia or distinguish different subtypes of dementia [152]. It does not assess executive functions, and there are only a few episodic and semantic memory or visuospatial tasks [153]. There are 11 items, with a maximum score of 30. For persons with at least eight years of education, the presence of suspected cognitive impairment or dementia is determined by a score below the cut-off value of 23/24 [136], with lower scores indicating increasing cognitive impairment [154]. Since 1975, numerous other cut-offs have been calculated from the receiver operating characteristic (ROC) curve analysis of specific populations together with adjustments of sociocultural variables (such as age, ethnicity, and education), which have been found to have an effect on the performance of the MMSE [155–157].

Six-Item Cognitive Impairment Test (6-CIT). The 6-CIT, originally referred to as Six-Item Orientation-Memory-Concentration Test, was developed in 1983 by Katzman and colleagues [158] by shortening the Blessed and colleagues' Mental Status Test [159]. It was designed as a screening test for dementia and is freely available. Because of its practicality, high acceptability, and decent psychometric properties [160], the 6-CIT has been used in research and a broad range of settings in clinical practice [161–165]. For use in primary care and hospital setting, the 6-CIT has been recommended as a cognitive screening tool by the Alzheimer's Society and the National Collaborating Centre for Mental Health (UK) [166, 167]. The 6-CIT takes less than 10 minutes to administer and involves three tests of temporal orientation, a short-term memory test and two tests of attention [160]. It is scored out of 28, scores greater than 10 indicate cognitive impairment [168]. Because of its verbal method of test administration, the 6-CIT can also be used in visually impaired patients [76]. The performance of the 6-CIT is influenced by age, education, and ethnicity [76, 160, 168] and thus needs adjustment when administered in diverse settings.

Time and Change Test (T&C). The T&C test, originally developed in 1998 by Inouye and colleagues, is a simple, standardized, performance-based test for the detection of dementia [126]. Due to its brevity, it takes less than five minutes to administer; it is highly acceptable to patients and may offer particular advantages in clinical and research settings where frequent examination of cognitive status is required [126, 169]. The T&C test incorporates supplemental cognitive domains such as calculation, conceptualization, and visuospatial ability [126]. It consists of a telling-time task and a making-change task. In the telling-time task, patients must respond to a clock face set at 11:10. Patients are allowed two tries within a 60-second period. If the patient fails to respond correctly, the task is terminated and recorded as an error. In the making-change task, the patients are asked to give one dollar in change from a group of coins with smaller denominations. Patients are allowed two tries in 120 seconds. Incorrect responses on either or both tasks indicate dementia. According to Inouye and colleagues, the T&C test is only minimally affected by education.

#### Diagnostic accuracy

**Fig 3:**
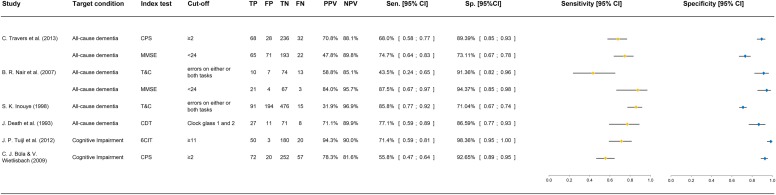
Summary of diagnostic accuracy data. (A) Abbreviations: CPS: Cognitive Performance Scale; MMSE: Mini-Mental Status Examination; 6-CIT: Six Item Cognitive Impairment Test; T&C: Time & Change test; CDT: Clock-Drawing Test (B) All 95% CI have been calculated using the Wilson formula with continuity correction.

The diagnostic accuracy data for the inpatient setting was extracted for each study and are summarized together with sensitivity, specificity, and statistical uncertainty intervals in the forest plot presented in Fig 3 (Summary of diagnostic accuracy data). Positive predictive values (PPV) and negative predictive values (NPV) are also summarized in the designated figure.

The study by Travers and colleagues applied the CPS and MMSE. For the CPS, at a threshold of ≥2, a sensitivity of 0.68 and specificity of 0.89 was reported [125]. The authors concluded, upon their study findings, that the CPS could substitute other widely used instruments for screening for dementia in older hospital inpatients. However, at present, the CPS can only be scored following administration of the complete interRAI Acute Care assessment. For the accuracy of the MMSE, which was evaluated as a comparator to the CPS, Travers and colleagues reported 0.75 for sensitivity and 0.73 for specificity using the common cut-off value of <24 [125]. The diagnosis according DSM-IV was established with access to the MMSE results. Therefore, overestimation is a legitimate concern.

For the accuracy of the 6-CIT, Tuijl and colleagues reported the largest number of correct positive predictions and the lowest number of false-positive predictions at a cut-off value of ≥11. Sensitivity and specificity were 0.71 and 0.98, respectively [124]. Compared with the MMSE, based on its equal performance but greater practicality, the 6CIT appears to be preferable to the MMSE in screening for cognitive impairment in older patients in a general hospital setting. However, the QUADAS-2 assessment revealed significant methodological limitations that may have led to overestimation of the accuracy of the 6-CIT.

The second study evaluating the diagnostic accuracy of the CPS, using the same common cut-off value of ≥2, reported 0.56 for sensitivity and 0.93 for specificity [122]. Compared to the rather positive conclusion of Travers and colleagues, the concluding Büla and colleague's statement remained more critical toward an implementation of the CPS in the hospital setting, mainly, because their analyses have shown that the CPS tends to underestimate cognitive impairment in older patients and overestimate it in dependent patients with depressive symptoms and comorbidities. Both are likely to be found in the hospital setting. Methodological limitations arose from the domain of patient selection, index test and reference standard. Due to inappropriate exclusions (privately insured patients and surgical patients), spectrum bias may have led to overestimation; thus, accuracy data should be interpreted with caution. Last, visual assessment of the forest plot showed high levels of heterogeneity where there is little overlap in the sensitivity confidence intervals of both CPS studies [122, 125].

For the T&C, which was evaluated in two studies, Inouye and colleagues reported 0.86 and 0.71 for sensitivity and specificity, respectively [126]. A positive test for either of the two components was classified as a positive result for dementia. According to the authors, the T&C was well tolerated and acceptable to nearly all participants across educational levels and diverse cultural backgrounds. Considering its relatively high sensitivity and negative predictive value (0.97), the T&C is advocated as a brief screener in high-risk settings for sequential use in combination with further clinical assessments to evaluate possible positive results. The methodological assessment showed no risk of bias or applicability concerns except for the index test, which was not administered early during hospitalization.

The second study with the objective to validate the T&C as a screening tool reported unusually divergent diagnostic accuracy values. For the same cut-off, Nair and colleagues reported in its Australian study 0.44 for sensitivity and 0.91 for specificity [121]. As evidenced by the high participation rate, the T&C test appeared, once again, to be readily acceptable but failed as a sensitive screening tool. As possible explanation, the authors argued that their country-specific adaptation of the making-change task, namely, the use of Australian coin money, may have varied the complexity and affected its sensitivity. The relatively small sample size (N = 103), accounted for low accuracy and large confidence intervals, especially with regard to sensitivity. Methodological limitations emerged from three of four domains. Only the patient selection domain was not rated as high risk of bias but was considered unclear because whether a consecutive or random sample was enrolled was not mentioned. Not surprisingly, the forest plot presented diverging confidence intervals without any overlap. For the second instrument evaluated, the MMSE, Nair and colleagues reported 0.88 for sensitivity and 0.94 for specificity [121]. Compared with the T&C, based on its superior performance and usefulness, the MMSE appears to be preferable to the T&C test in screening for dementia in older, general-hospital inpatients. However, in addition to the above mentioned methodological limitations, another potential problem with this study was that the DSM-IV diagnoses and the MMSE scores were obtained by the same interviewer. This possible source of confounding may have led to an overestimation of the performance of the MMSE relative to the T&C test. In comparison to the MMSE accuracy data presented by Travers and colleagues, the visual assessment of the forest plot showed, at least for the sensitivity confidence intervals, a good degree of overlap at a cut-off value of <24.

Last, for the CDT, Death and colleagues presented 0.77 and 0.87 for sensitivity and specificity, respectively [123]. Because of its characteristics, the test is proposed as a simple and rapid tool for use by admitting junior staff to highlight possible dementia and to alert to the necessity for further testing. The QUADAS-2 assessment revealed methodological limitations regarding the flow and timing domain that may have led to overestimation or underestimation of the accuracy of the CDT. In particular, because patients were only reviewed according to DSM-III diagnostic criteria, if a discrepancy in test results occurred between the CDT and MMSE, accuracy should be interpreted with caution. Furthermore, the reference standard domain was considered to be at unclear risk because whether the reference standard results were interpreted with or without the knowledge of the results of the index test was not stated. Finally, the comparative visual assessment revealed rather large confidence intervals, which may originate from the relatively small study sample (N = 117).

Because of the insufficient number of included studies, no meta-analysis of the diagnostic test accuracy, investigation of heterogeneity, and sensitivity analyses were conducted.

## Discussion

### Summary of main results

The aim of this review was to search for and identify adequate instruments for screening for dementia and MCI in unselected, elective hospital inpatients, with a restriction to validation studies of high quality to minimize possible biases due to methodological shortcomings and differences in reporting. Accordingly, this review applied a well-constructed search strategy and included quality assessments. The overall number of studies included in this review is small. Only six studies evaluating five unique screening instruments were found. Four instruments, the Cognitive Performance Scale (CPS), the Mini-Mental Status Examination (MMSE), the Time & Change (T&C), and the Clock Drawing Test (CDT) were investigated as screening tools for detecting dementia; the CPS and the 6-Item Cognitive Impairment Test were investigated as screening tools for unspecified cognitive impairment. Overall, despite a restrictive combination of inclusion criteria, a considerable number of included studies were rated as having a high risk of bias or applicability concerns, in particular in the flow and timing and index test domain, respectively. In addition, in six cases, risk of bias was rated as unclear. This originated directly from the limitations in reporting, when older articles seemingly adhered less to recent guidelines. Consequently, the scarcity of information, methodological limitations, and heterogeneity of study characteristics did not allow formal meta-analyses of study results and further analysis.

### Strengths and weaknesses of the review

The strengths of this review include the use of a multi-concept search strategy to identify a wide spectrum of potential articles, which would reduce the risk of publication bias. The primary search concept used terms from four domains and was complemented by a more rigorous second concept, which used instrument names from the Dementia Practice Guidelines as key search terms. While the latter concept ensured coverage of widely used instruments, primarily in the primary care setting, the former targeted, but more sensitive search approach, may have identified studies that could have been overlooked. For quality and comprehensiveness reassurance, the search strategy was peer-reviewed, using the PRESS 2015 Guideline Evidence-Based Checklist.

Importantly, this review also included a detailed quality assessment that provided crucial information for the interpretation of the reported studies. For quality assessment, the recommended assessment tools for Diagnostic Accuracy Studies (QUADAS-2) and the Standards for the Reporting of Diagnostic Accuracy studies (STARD 2015) checklist were applied. This review itself reports according to the PRISMA Statement for Preferred Reporting Items for a Systematic Review and Meta-Analysis of Diagnostic Test Accuracy Studies (PRISMA-DTA statement 2018).

Finally, in contrary to recent reviews, this systematic review excluded case-control studies, which are prone to overestimate diagnostic accuracy by including phenotypic extremes; that is two extreme populations are compared, rather than typical healthy and diseased populations. Complementary, the focus on rather naturalistic, cross-sectional validation studies provided an applicable choice of instruments for systematic screening during hospital routine care.

This review has several limitations. Formal meta-analyses and additional analysis were precluded due to the small number of studies reported. Initially, for meta-analysis of sensitivity and specificity, it was planned to use the bivariate random-effects model approach (if studies used the same index test at a common threshold) or the hierarchical summary ROC (HSROC) method (if multiple thresholds were reported) [170, 171]. For the investigation of heterogeneity, in addition to the visual examination of the forest plot, performing meta-regression was planned by fitting HSROC models with pre-specified covariates. Therefore, drawing conclusions from the reported studies regarding the diagnostic accuracy of included screening instruments was limited.

Furthermore, the inclusion of the MMSE as a criterion standard could be criticized. The choice to accept any screening instrument as a criterion standard could be justified by two reasons: (1) In particular in cross-sectional studies embedded in daily clinical routine, the confirmation of the index test with a more adequate reference standard, i.e. clinical assessment, or neuropsychological testing, with explicit diagnostic criteria with or without expert consensus, is usually logistically constrained and thus, often deliberately avoided. (2) The diagnostic criteria for MCI used in this review are relatively recent. Therefore, with the motive to increase the number of potentially eligible studies, this review also included diagnostic accuracy studies comparing their index test with the MMSE as a criterion standard. The choice of the MMSE is justified by its widespread use in research and popularity in the clinical setting. However, even though the MMSE is widely used, it has imperfect specificity and sensitivity, and very limited ability to differentiate between MCI patients and healthy controls [154].

An additional limitation originated from low methodological quality of some included studies. Instrument accuracy was potentially overestimated (due to selection bias, time lag bias, information bias, and study result elimination bias); thus, results should be interpreted with caution. The exclusion of informant-rated questionnaires, web-based and telephonic screening tools can be seen as another shortcoming. Due to this restriction, some promising screening instruments were not evaluated in this review. Finally, the exclusion of non-English language, gray literature, and unpublished studies also had potential for bias.

### Applicability of findings to the review question

In 2013, Jackson and colleagues conducted a very similar review and meta-analysis [86]. Their intent was to determine which of the instruments advocated for screening for dementia had been validated in older hospital inpatients. In the end, in most of their included studies, the sample population was either mixed with outpatients [172, 173], geriatric [174–176], or admitted through the emergency department [177, 178]. Jackson and colleagues reported the largest evidence base (with more than one report) for the use of the Abbreviated Mental Test Score (AMTS) and stated a clear need for more validation studies to inform screening for dementia in hospital inpatients best. In 2018, Carpenter and colleagues performed a systematic review and meta-analysis of the diagnostic accuracy of brief screening instruments for dementia in geriatric ED patients [82]. The AMT-4, a shorter, 4-item version of the AMTS, was found the most accurate ED screening instrument to rule in dementia.

This present review found only a small number of validated instruments and was not able to recommend a single best instrument. Concerning the AMTS, for screening MCI or dementia in unselected, elective hospital inpatients, this review found no evidence. Although the findings of this review do not advocate a specific instrument in terms of best diagnostic accuracy, the results do suggest that for screening dementia there are valuable instruments as the majority of the included studies report satisfying sensitivity and negative predictive values–both of which need to be maximized in order to miss relatively few true cases.

The lack of evidence is surprising, because despite the wider public interest in dementia and the recent debates about targeted screening initiatives, this review found not one eligible study published after 2013. Although it was not possible to estimate the pooled operating characteristics, the included description of instrument characteristics, the descriptive analysis of performance measures, and the critical evaluation of the reporting studies may contribute to clinicians' choice of the best screening instrument for their purpose.

### Implications for clinical practice

At the present time, there is insufficient evidence to recommend for or against the use of a specific instrument for screening for dementia or MCI in older hospital inpatients. Although some instruments performed comparatively well and were advocated by the individual study authors, based on the limited information currently available, a universal recommendation for routine use would be of questionable quality and little clinical utility. In the end, whatever test is used, evidence of cognitive impairment on single tests must be interpreted in the light of contextual and other information. The main caveat is that simple cognitive tests used in isolation are not reliable enough. In addition, even in the absence of dementia, inpatients may perform poorly because of other reasons (e.g., medication, pain, language barriers, and cultural issues) and/or competing disorders (e.g., delirium, depression, diabetes) [179]. Delirium is the most common cause; it affects at least one in 10 hospital inpatients [79, 180, 181]. For this reason, a sequential use in combination with detailed expert assessments is highly recommended before establishing diagnosis and following care pathways.

If screening is chosen, timing matters. In general, the sooner MCI or dementia is identified during a hospital stay, the sooner appropriate interventions can be tailored to the individual's needs (e.g., choice of anesthesia, involvement of primary caregiver, medication management) [94]. Under the assumption that elective inpatients are in general more stable and are not in need of immediate care, clinicians should consider incorporating screening as part of the overall hospital admission assessment and follow-up further evaluations both during and after hospitalization. In many cases, subsequent detailed examinations may only be realistic after discharge. As long as clinicians are accustomed to managing possible confounding factors and are trained in the use of cognitive tests, such test do have a role in screening for dementia or MCI in hospital inpatients [90]. However, the time needed to perform assessments of cognitive function means an increase in the workload. Although the costs of initial screening can be kept quite inexpensive, the costs of a subsequent diagnostic workup will vary, depending on the specific diagnostic pathway. Finally, it needs to be said that at present, evidence that screening for dementia is effective is lacking [41, 182].

### Implications for research

At present, there is a clear need for further validation studies of dementia or MCI screening instruments for older hospital inpatients, rather than for the development of new instruments. Future studies should incorporate strong methodological study designs to minimize the risks of bias but also need to report in sufficient detail, so that trustworthiness and applicability of the study findings can be judged. The conduct of a meta-analysis might be a valuable objective for future research, provided that the number of validation studies to be evaluated is sufficient. In addition, on the basis of well-validated cognitive tests, distinct recommendations for clinicians on how to identify patients with possible dementia systematically should be established. Ultimately, this will also require additional evidence regarding the cost-effectiveness of screening for dementia or MCI. A corresponding analysis of the benefits and costs of screening should be measured in terms of the value of timely and correct diagnosis and the application of adequate medical treatments and care management programs.

## Supporting information

S1 Appendix Search strategy for PubMed. (PDF)

S2 Appendix Assessment of methodological quality using QUADAS-2. (PDF)

S3 Appendix Standards for the Reporting of Diagnostic Accuracy studies checklist. (PDF)

S4 Appendix Study protocol. (PDF)

S5 Appendix Prisma 2009 checklist. (PDF)

S6 Appendix Minimal data set. (XLSX)
