# Supplementary material for: Validity of screening instruments for the detection of dementia and mild cognitive impairment in hospital inpatients: A systematic review of diagnostic accuracy studies
Source: PLoS One. 2019 Jul 25;14(7):e0219569. doi: 10.1371/journal.pone.0219569 (PMC6657852; doi:10.1371/journal.pone.0219569)
Supplement: S1 Appendix — (PDF) [file pone.0219569.s001.pdf]

S1. Appendix

Search strategy for PubMed

SETTING

	Number	Search Term	Findings
1		Aged[MeSH Terms]	2395350
2		Inpatients[MeSH Terms]	6786
3		Hospitalization[MeSH Terms]	79284
4		Hospitals [MeSH Terms]	38484
5		Inpatient[tw] OR Inpatients[tw]	31741
6		Hospitalization[tw]	57873
7		Hospitals[tw] OR Hospital[tw]	265366
8		Preoperative Period[MeSH Terms]	3056
9		Preoperative Care[MeSH Terms]	16724
10		Elective Surgical Procedures[MeSH Terms]	5151
11	1 AND (2 – 10 (OR))	(((Inpatients[MeSH Terms] OR Hospitalization[MeSH Terms] OR Hospitals [MeSH Terms] OR Inpatient[tw] OR Inpatients[tw] OR Hospitalization[tw] OR Hospitals[tw] OR Hospital[tw] OR Preoperative Period[MeSH Terms] OR Preoperative Care[MeSH Terms] OR Elective Surgical Procedures[MeSH Terms]))) AND Aged[MeSH Terms]	333855

DISEASE

	Number	Search Term	Findings
1		Dementia[MeSH Terms]	62443
2		Cognition Disorders[MeSH Terms]	28962
3		cognition[Title] OR cognitive[Title] OR dementia[Title] OR Alzheimer*[Title]	61238
4	1 – 3 (OR)	(Dementia[MeSH Terms] OR Cognition Disorders[MeSH Terms] OR cognition[Title] OR cognitive[Title] OR dementia[Title] OR Alzheimer*[Title])	86042

OUTCOME

	Number	Search Term	Findings
1		Sensitivity and specificity[MeSH Terms]	144745
2		Predictive value of tests[MeSH Terms]	73030
3		Validation[tw] OR Validate[tw] OR validity[tw]	63206
4		Psychometrics[MeSH Terms]	17329
5		Prevalence[MeSH Terms]	73523
6	1- 5 (OR)	(Sensitivity and specificity[MeSH Terms] OR Predictive value of tests[MeSH Terms] OR Validation[tw] OR Validate[tw] OR validity[tw] OR Psychometrics[MeSH Terms] OR Prevalence[MeSH Terms])	268129

INTERVENTION

	Number	Search Term	Findings
1		Diagnostic tests, routine[MeSH Terms]	1823
2		Mass screening[MeSH Terms]	22235
3		Early diagnosis[MeSH Terms]	11961
4		Neuropsychological Tests[MeSH Terms]	50210
5		Geriatric Assessment[MeSH Terms]	21583
6		Intelligence Tests[MeSH Terms]	2488
7		Psychiatric Status Rating Scales[MeSH Terms]	23256
8		Screen[tw] OR Screening[tw]	80365
9		test[Title]	16192
10		tests[Title]	5071
11		Tool[Title]	5332
12		Tools[Title]	1087
13		Measures[Title]	5942
14		Measure[Title]	4226
15		Instrument[Title]	1939
16		Instruments[Title]	871
17		Assessment[Title]	38332
18		Detection[Title]	23021
19	1 – 18 (OR) OR instruments recommended in primary care dementia practice guidelines	((5 word test[tw] OR Five word test[tw] OR 6 item cognitive impairment test[tw] OR Six item cognitive impairment test[tw] OR 6CIT[tw] OR 7 Minute Screen[tw] OR Seven Minute Screen[tw] OR Abbreviated mental test[tw] OR Blessed Orientation Memory Concentration Test[tw] OR BOMC[tw] OR BrainCheck[tw] OR Clock Drawing test[tw] OR Elderly Cognitive Assessment Questionnaire[tw] OR ECAQ[tw] OR ECDR[tw] OR Expanded Clinical Dementia Rating[tw] OR Frontal Assessment Battery[tw] OR GPCOG[tw] OR General Practitioner Assessment of Cognition [tw] OR Hasegawa Dementia Scale[tw] OR Mattis Dementia Rating Scale[tw] OR Mini Cog[tw] OR MiniCog[tw] OR MMSE[tw] OR Mini-Mental State Examination[tw] OR Modified MMSE[tw] OR Modified-Mini-Mental State Examination[tw] OR MOCA[tw] OR Montreal Cognitive Assessment[tw] OR Saint Louis University Mental Status Examination[tw] OR St. Louis University Mental Status Examination[tw] OR SLUMS[tw])) OR (Diagnostic tests, routine[MeSH Terms] OR Mass screening[MeSH Terms] OR Early diagnosis[MeSH Terms] OR Neuropsychological Tests[MeSH Terms] OR Geriatric Assessment[MeSH Terms] OR Intelligence Tests[MeSH Terms] OR Psychiatric Status Rating Scales[MeSH Terms] OR Screen[tw] OR Screening[tw] OR test[Title] OR tests[Title] OR Tool[Title] OR Tools[Title] OR Measures[Title] OR Measure[Title] OR Instrument[Title] OR Instruments[Title] OR Assessment[Title] OR Detection[Title])	232501

Instruments recommended for dementia screening in Primary Care setting were extracted from the following Dementia Practice Guidelines, if the administration time < 15 min, included more than one cognitive domain and were not only targeting informants.

Dementia Practice Guideline	Recommended instruments (brief cog. testing instrument)
Ministry of Health Singapore (2013)	Elderly Cognitive Assessment Questionnaire, Abbreviated Mental Test, AD8, Mini Mental State Examination, Frontal Assessment Battery, Montreal Cognitive Assessment
European Federation of Neurological Sciences (2012)	Mini-Mental State Examination (MMSE), 7min, Addenbrooke´s cognitive examination revised(ACE-R), MOCA, Mattis Dementia Rating Scale (MDRS), Clock Drawing test (CDT), 5 word test,
Consensus 2012 - Diagnosis and Treatment of Patients with Dementia in Switzerland	Mini-Mental Status Examination, Clock Drawing Test, MOCA, Minicog, BRAINCHECK,
National Institute for Health and Clinical Excellence (2011)	MMSE, 6-item Cognitive Impairment Test (6-CIT), General Practitioner Assessment of Cognition (GPCOG), 7-Minute Screen
Clinical Research Centre for Dementia of South Korea (2011)	Mini-Mental State Examination, Expanded Clinical Dementia Rating, Global Deterioration Scale, Modified-MMSE, Hasegawa Dementia Scale, 7-Minute Screen Test, Montreal Cognitive Assessment
California Workgroup on Guidelines (2011)	Blessed Orientation Memory Concentration Test (BOMC), Cognitive Assessment Screening Instrument (CASI), MiniCog, Mini-Mental State Examination, MOCA, St. Louis University Mental Status Examination (SLUMS)

Final search string for PubMed

Setting 11 AND Intervention 19 AND Disease 4 AND Outcome 6	904

Filters

Database: Pubmed
Access Date: 04.10.2019
Filters:

- **Article Type** included: Clinical trial, clinical study, controlled clinical trial, journal article, meta-analysis, multicentre study, observational study, randomized controlled trial, review, systematic review, validation study
- **Language** selected: English & German,
- **Age** included: 65+ years

Findings before filters: 954, **after:** 904
Time range of findings: 1972-2019
Abbreviations: MeSH = Medical Subject Headings, TW = Text words
