# Supplementary material for: Validity of screening instruments for the detection of dementia and mild cognitive impairment in hospital inpatients: A systematic review of diagnostic accuracy studies
Source: PLoS One. 2019 Jul 25;14(7):e0219569. doi: 10.1371/journal.pone.0219569 (PMC6657852; doi:10.1371/journal.pone.0219569)
Supplement: S2 Appendix — (PDF) [file pone.0219569.s002.pdf]

S3. Appendix

Assessment of methodological quality using QUADAS-2

Domain	Participant Selection	Index Test	Reference Standard	Flow and Timing
Description	Describe methods of participant selection: Describe included participants (prior testing, presentation, intended use of index test and setting)	Describe the index test and how it was conducted and interpreted	Describe the reference standard and how it was conducted and interpreted	Describe any participants who did no receive the index test and or reference standard or who were excluded from the 2x2 table (refer to flow diagram): Describe the time interval and any interventions between index test and reference standard
Signaling questions (yes/no/unclear)	Was a consecutive or random sample of participants enrolled?	Were the index test results interpreted without knowledge of the results of the reference standard?	Is the reference standard likely to correctly classify the target condition?	Was there an appropriate interval between index test(s) and reference standard?
	Was a case-control design avoided?	If a threshold was used, was it pre-specified?	Were the reference standard results interpreted without knowledge of the results of the index test?	Did all participants receive a reference standard?
	Did the study avoid inappropriate exclusions?			Did all participants receive the same reference standard?<br>Were all participants included in the analysis?
Risk of bias: (High/low/ unclear)	Could the selection of participants have introduced bias?	Could the conduct or interpretation of the index test have introduced bias?	Could the reference standard, its conduct, or its interpretation have introduced bias?	Could the participant flow have introduced bias?
Concerns regarding applicability: (High/low/ unclear)	Are there concerns that the included participants do not match the review question?	Are there concerns that the index test, its conduct, or interpretation differ from the review question?	Are there concerns that the target condition as defined by the reference standard does not match the review question?	
