# Supplementary material for: Validity of screening instruments for the detection of dementia and mild cognitive impairment in hospital inpatients: A systematic review of diagnostic accuracy studies
Source: PLoS One. 2019 Jul 25;14(7):e0219569. doi: 10.1371/journal.pone.0219569 (PMC6657852; doi:10.1371/journal.pone.0219569)
Supplement: S3 Appendix — (PDF) [file pone.0219569.s003.pdf]

S4. Appendix

Standards for the Reporting of Diagnostic Accuracy studies checklist

Section	No	Item
**Title or abstract**		
	1	Identification as a study of diagnostic accuracy using at least one measure of accuracy (such as sensitivity, specificity, predictive values or AUC)
**Abstract**		
	2	Structured summary of study design, methods, results and conclusions (for specific guidance, see STARD for Abstracts)
**Introduction**		
	3	Scientific and clinical background, including the intended use and clinical role of the index test
	4	Study objectives and hypotheses
**Methods**		
Study Design	5	Whether data collection was planned before the index test and reference standard were performed (prospective study) or after (retrospective study)
Participants	6	Eligibility criteria
	7	On what basis potentially eligible participants were identified (such as symptoms, results from previous tests, inclusion in registry)
	8	Where and when potentially eligible participants were identified (setting, location and dates)
	9	Whether participants formed a consecutive, random or convenience series
Test methods	10a	Index test, in sufficient detail to allow replication
	10b	Reference standard, in sufficient detail to allow replication
	11	Rationale for choosing the reference standard (if alternatives exist)
	12a	Definition of and rationale for test positivity cut-offs or result categories of the index test, distinguishing pre-specified from exploratory
	12b	Definition of and rationale for test positivity cut-offs or result categories of the reference standard, distinguishing pre-specified from exploratory
	13a	Whether clinical information and reference standard results were available to the performers or readers of the index test
	13b	Whether clinical information and index test results were available to the assessors of the reference standard
Analysis	14	Methods for estimating or comparing measures of diagnostic accuracy
	15	How indeterminate index test or reference standard results were handled
	16	How missing data on the index test and reference standard were handled
	17	Any analyses of variability in diagnostic accuracy, distinguishing pre-specified from exploratory
	18	Intended sample size and how it was determined
**Results**		
Participants	19	Flow of participants, using a diagram
	20	Baseline demographic and clinical characteristics of participants
	21a	Distribution of severity of disease in those with the target condition
	21b	Distribution of alternative diagnoses in those without the target condition
	22	Time interval and any clinical interventions between index test and reference standard
Test results	23	Cross tabulation of the index test results (or their distribution) by the results of the reference standard
	24	Estimates of diagnostic accuracy and their precision (such as 95% CIs)
	25	Any adverse events from performing the index test or the reference standard
**Discussion**		
	26	Study limitations, including sources of potential bias, statistical uncertainty and generalizability
	27	Implications for practice, including the intended use and clinical role of the index test
**Other information**		
	28	Registration number and name of registry
	29	Where the full study protocol can be accessed
	30	Sources of funding and other support; role of funders
