# Supplementary material for: Validity of screening instruments for the detection of dementia and mild cognitive impairment in hospital inpatients: A systematic review of diagnostic accuracy studies
Source: PLoS One. 2019 Jul 25;14(7):e0219569. doi: 10.1371/journal.pone.0219569 (PMC6657852; doi:10.1371/journal.pone.0219569)
Supplement: S4 Appendix — (PDF) [file pone.0219569.s004.pdf]

S5. Appendix

Study protocol

1. Administrative information

1.1. Title

Cognitive tests for the detection of possible Alzheimer's disease and other dementias in hospital inpatients:

Study protocol of a systematic review

1.2. Registration

Not applicable/registered

1.3. Authors

1.3.1. Contact

Aljoscha B. Hwang (ABH)
Stab Medizin
Luzerner Kantonsspital
Spitalstrasse, 6000 Luzern 16

Aljoscha.b.hwang@hotmail.com
Corresponding Author

Stefan Boes (SB)
Department of Health Sciences and Health Policy
Universität Luzern
E-Mail: Stefan.Boes@unilu.ch

Thomas Nyffeler (TN)
Clinic for Neurology and Neurorehabilitation
Luzerner Kantonsspital
E-Mail: Thomas.Nyffeler@luks.ch

1.3.2. Contributions

Aljoscha B. Hwang (ABH)	Stefan Boes (SB)	Thomas Nyffeler (TN)
Conceptualization, Methodology, Investigation, Writing	Conceptualization, Methodology, Resources, Supervision, Writing (Reviewer)	Conceptualization, Methodology, med. Supervision, Writing (Reviewer)

1.4. Amendments

Not applicable

1.5. Support

1.5.1. Sources

Not applicable

1.5.2. Sponsor

Not applicable

1.5.3. Role of sponsor or funder

Not applicable

2. Introduction

2.1. Rationale

Despite the absence of a cure for dementia, numerous dementia strategies emphasize earlier diagnosis and intervention. This drive toward earlier diagnosis and intervention has been accompanied by a debate about the value of arriving at a diagnosis of dementia earlier in the disease process. Several studies reported evidence that supports a possible beneficial effect of early and accurate diagnosis. As a result, changes in health care policies and priorities, such as the introduction of an opportunistic "dementia case-finding scheme" in the United Kingdom, or the implementation of cognitive assessments in the Medicare Annual Wellness Visit in the United States have occurred.

Country-specific guidelines and/or systematic literature reviews on which instruments to favor have already been published for the primary care setting. For the hospital setting however, where dementia and cognitive impairment are much more prevalent comparable guidelines do not exist.

Even though screening instruments advocated for the use in primary care setting are often not restricted to primary care, variations in demographic features, disease prevalence, and severity but also, differences in test conditions (e.g., timing, interventions between index test and reference standard) entail external validation prior to general application in hospital setting. In response to this demand, two systematic reviews have been conducted to establish adequate tools for dementia screening, considering the particularities of the population to be evaluated. However, while B.A. Appels et al. (2010) mostly reported validation studies sampling from selected outpatients with a focus on rather extensive screening instruments (10 to 45min administration time), Jackson et al. (2013) found a remarkable lack of robust evidence; the largest evidence base was found for the use of the Abbreviated Mental Test Score (AMTS), and reported a clear need for more validation studies to inform screening for dementia in hospital inpatients best.

In the hospital setting, the knowledge that a patient has or might have dementia is essential because of the multiple immediate implications for care. Hospital medical staff may administer brief cognitive screening tests before or on the day of admission and, depending on the test results, cause additional investigations to be made to confirm whether a diagnosis is present; provide appropriate care during the hospital stay (e.g., choice of anesthesia, involvement of primary caregiver, medication management, etc.), and realize adequate discharge management, which may then lead to avoiding new medical events known to be more likely among patients with dementia and promoting earlier diagnosis.

2.2. Objectives

Many screening instruments are recommended for the application in primary care setting but not so many, for screening in older, unselected hospital inpatients. The aim of this review is to provide clinicians, who wish to implement dementia screening, an up-to-date choice of practical and accurate instruments that have been validated well for use in older hospital inpatients.

3. Methods

3.1. Eligibility criteria

Language: English and German

Study size: >100

Target condition: Mild cognitive impairment, dementia, and any common dementia subtype

Setting: General or university hospital; medical and surgical wards

Population: Unselected samples of elective inpatients (male and female) older than 64 years; mixed cohorts only if separate reporting of data or inpatients form majority of sample

Index test: Multi-domain, brief (<15min), performance test, excluding informant rated, telephonic or computerized tests, excluding measures, assessing daily living activities and functional status, excluding self-administered tests

Reference Standard: Any version of the Diagnostic and Statistical Manual of Mental Disorders (DSM), any version of the International Classification of Diseases (ICD), expert diagnosis following interview, or Mini-Mental State Examination (MMSE) application as criterion standard.

Study design: Cross-sectional studies, in which the index test and reference standard were performed during the hospital stay, excluding case-control and longitudinal studies, excluding studies relying on post-mortem verification of neuropathological diagnoses

3.2. Information sources

Databases: PubMed, Cochrane library and PsycINFO (+ systematic reviews, reference sections and similar article feature in PubMed); excluding grey literature

Date coverage: All published articles

Contact with study authors: Max three attempts via Email in order to resolve issues e.g., missing data, lack of clarity, etc.

3.3. Search strategy for PubMed

See Appendix S1. Search strategy for PubMed

3.4. Data management

For literature management (Citations, abstracts, full-texts) EndNote will be used.

3.5. Selection process

Screening (titles and abstracts) and full-text assessment will be performed by two independent reviewers using eligibility criteria. Disagreements will be decided by consensus.

3.6. Data collection process

Verify data extraction by at least one reviewer – decide by consensus. Contact authors in case of uncertainties (Mail).

3.7. Data items

Extract country; type of hospital; patient group; target condition; sample size; age, and mean age; gender ratio; level of education; index test and applied cut-off; reference standard; point in time of screening; other assessments; assessment for delirium; prevalence, and accuracy data (2x2 table, sensitivity, specificity, etc. will be calculated) using piloted extraction sheet.

Extract all data required for quality assessments (QUADAS2).

General instrument characteristics (Instrument name, method of administration, administration time, availability, cognitive functions covered, advantages and disadvantages)

3.8. Outcomes and prioritization

1) Study characteristics

2) Accuracy data
3) QUADAS2
4) STARD
5) General instrument characteristics

3.9. Risk of bias in individual studies

Two reviewers will independently assess, discuss and reach consensus on the methodological quality of all included studies (QUADAS2 and STARD 2015).

3.10. Data synthesis

Statistical analysis will be performed according to the Cochrane guidelines for diagnostic test accuracy reviews. Diagnostic accuracy data will be presented in a 2x2 table. Based on the 2x2 table, sensitivity and specificity values as well as measures of statistical uncertainty will be calculated. Diagnostic accuracy data will be plotted on a coupled forest plot. Only sensitivity and specificity data at the most common threshold will be included.

For meta-analysis of sensitivity and specificity, bivariate random-effects model approach (if studies use the same index test at a common threshold) or the hierarchical summary ROC (HSROC) method (if multiple thresholds are reported) will be used. For the investigation of heterogeneity, in addition to the visual examination of the forest plot, meta-regression will be done by fitting HSROC models with pre-specified covariates (e.g., baseline prevalence, reference standard, quality criteria from QUADAS2 assessment).
